# ESTRO guidelines for developing questionnaires in survey-based radiation oncology research

**DOI:** 10.1016/j.ctro.2024.100895

**Published:** 2024-11-24

**Authors:** Amanda Webster, Lotte S. Fog, Emma Hall, Peter S.N. van Rossum, Daan Nevens, Pierre Montay-Gruel, Pierfrancesco Franco, Elizabeth Joyce, Nuria Jornet, Catharine H. Clark, Jenny Bertholet

**Affiliations:** a Cancer Division, University College London Hospital (UCLH), London, United Kingdom; b Department of Medical Physics and Biomedical Engineering, University College London (UCL), London, United Kingdom; c National Radiotherapy Trials Quality Assurance (RTTQA) Group, University College Hospital (UCLH), United Kingdom; d Alfred Health Radiation Oncology, Melbourne, Victoria, Australia; e The Ocular Oncology Clinic, The Royal Victorian Eye and Ear Hospital, Melbourne, Victoria, Australia; f Clinical Trials and Statistics Unit, The Institute of Cancer Research, London, United Kingdom; g Department of Radiation Oncology, Amsterdam UMC, Location VUmc, Amsterdam, the Netherlands; h Iridium Netwerk, Radiotherapy Department, Antwerp REsearch in Radiation Oncology (AReRO), Center for Oncological Research (CORE), Integrated Personalized and Precision Oncology (IPPON), University of Antwerp, Antwerp, Belgium; i Department of Translational Medicine (DIMET), University of Eastern Piedmont, Novara, Italy; j Department of Radiation Oncology, ’Maggiore della Carità’ University Hospital, Novara, Italy; k Radiotherapy Department, Royal Marsden Hospital, Surrey, United Kingdom; l Servei de Radiofisica i Radioproteccio, Hospital de la Santa Creu i Sant Pau, Barcelona, Spain; m Radiotherapy Physics, University College London Hospital, London, UK; n Division of Medical Radiation Physics and Department of Radiation Oncology, Inselspital, Bern University Hospital, and University of Bern, Bern, Switzerland

**Keywords:** Guidelines, Survey-based research, Questionnaires, Radiation oncology

## Abstract

Survey-based research is increasingly used in radiation oncology, yet survey-based research methodology is often unfamiliar in the field. This guideline offers comprehensive instructions for conducting survey-based research in radiation oncology, covering critical aspects such as survey design, validation, dissemination, analysis, and reporting. Tailored to professionals, it emphasizes the importance of methodological rigour to ensure reliable and actionable data collection. Dissemination strategies are highlighted to maximize response rates and enhance data completeness across diverse clinical, research and industrial settings. Rigorous analysis techniques are discussed to uncover insights that optimize operational efficiencies and inform evidence-based practices. Transparent reporting is underscored as crucial for enhancing the credibility and applicability of findings. This guideline aims to be a practical resource for enhancing research standards in survey-based research for researchers and practitioners in radiation oncology.

## Highlights

• Clear survey design generates relevant data from radiation oncology staff and centres.
• Validation helps ensure that survey results are reliable and applicable to radiation oncology practices.
• Strategic dissemination maximizes response rates among professionals and radiation oncology centres.
• Planning analysis from the start ensures targeted and actionable insights in radiation oncology.
• Transparent reporting enhances the credibility and applicability of findings.

## 1 Introduction

Survey-based research in radiation oncology (RO) is widely used as a valuable tool to evaluate the implementation of novel radiotherapy technologies [1], [2], [3], [4], [5], examine practice patterns [6], [7], [8], [9], assess patient care [10], gauge the opinions or perspectives of RO staff on professional matters [11], [12], and assess the needs for education and training at the personal or national level [13], [14], [15], [16], [17], [18]. RO is a multifaceted interprofessional field encompassing a range of specialities from healthcare providers to researchers. These professionals include − but are not limited to- biologists, clinicians, nurses, physicists, radiation therapists, biomedical engineers, and biostatisticians operating within diverse settings such as clinical practice, research environments, industry, or a combination thereof. Consequently, generic survey-based research guidelines may be tricky to apply to research questions within the field of RO. Introducing specific guidelines and standardising surveys in RO could support good research practices by improving consistency, comparability, collaboration, and overall quality. Additionally, employing robust survey-based research methodologies could improve the validity of findings, support evidence-based practice, facilitate effective communication, and optimize resource allocation. This guideline provides a comprehensive and practical resource to assist RO researchers and professionals in developing questionnaires to use in survey-based research.

Tailored toward the specific needs of RO, the recommendations within this guideline focus on surveying institutional-level practices, implementation patterns, and individual-level professional attitudes and opinions including matters of education and training. The emphasis is deliberately placed on addressing research questions pertinent to institutions, RO professionals or scientific societies. Although important, the guideline deliberately excludes the development of questionnaires intended for patients (i.e. patient-reported outcomes, experience, or quality-of-life questionnaires). Designing such questionnaires entails distinct considerations beyond this guideline's scope, and specific research is addressing this [19], [20], [21].

Survey-based research relies on the same fundamental principles as any other type of research, starting with clearly defining the research question and gathering high-quality data. The choice to employ a survey to answer the research question should be intentional and consider the strengths and limitations of questionnaires as well as other possible methodologies [22].

This guideline focuses on survey methodology to gather high-quality data. RO researchers may be unfamiliar with these types of methods, and therefore examples are provided where appropriate. Templates for cover letters and specific recurring questions are provided (Appendix A.1.), aiming to assist researchers in questionnaire design and standardising general aspects for improved comparability in the field of RO surveys. This standardisation will also facilitate the tracking of practice changes over time. Specific aspects of design, validation, dissemination, analysis, and reporting are discussed, and a checklist is provided (Appendix A.2.) for RO researchers and professionals embarking on a survey-based research project. A glossary of survey terminology can be found in Appendix A.3.

This guideline is designed as a practical guide, to assist researchers in conducting high-quality survey-based research that aligns with the standards for publication in ESTRO journals. It is intended to be followed by researchers in their effort to obtain ESTRO support and/or endorsement for future survey-based research projects.

## 2 Material and methods

This guideline was synthesized by combining knowledge from 1) *general* guidelines pertaining to survey-based research (non-specific to the field of RO); 2) *RO-specific* literature using survey-based research; and 3) the authors’ own expertise gained through survey-based research within the field of RO. The ensuing recommendations were categorized into five principal themes: design, validation, dissemination, analysis, and reporting.

### 2.1 General guidelines for survey-based research

A literature search was conducted to identify general guidelines on survey-based research encompassing all five themes listed above. The search was neither systematic nor structured. Initially, each of the authors conducted independent searches for general survey guidelines. References in these papers were also screened and included if relevant. The findings were discussed collectively among all authors. The search for papers continued until all authors agreed that the information had reached a point of saturation, meaning no new insights were identified.

### 2.2 RO-specific publications using survey-based research

**Fig. 1:**
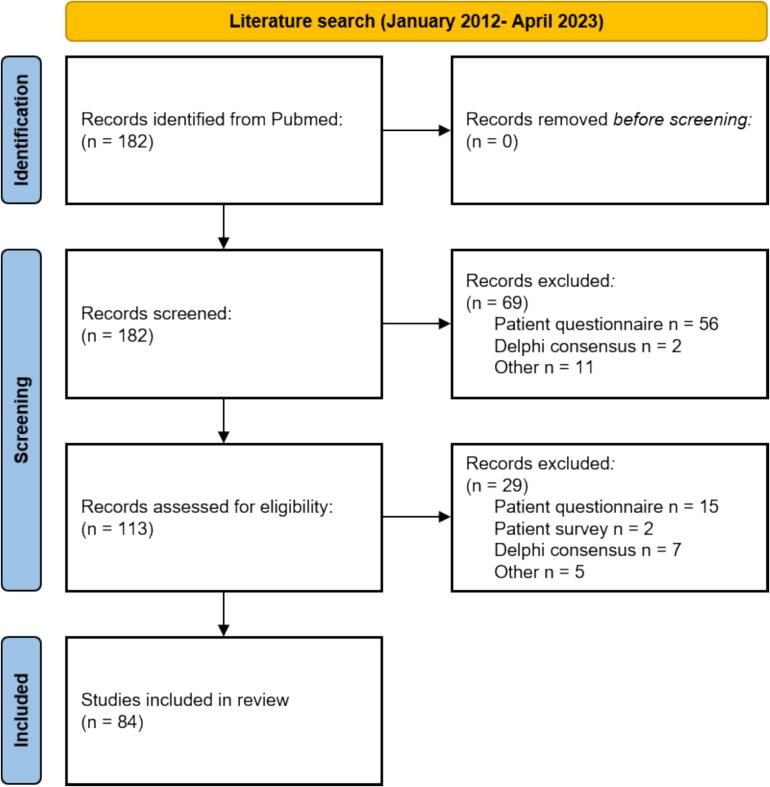
PRISMA diagram of the literature search for survey-based research papers in the ESTRO journals.

A scoping review, following PRISMA principles [23], [24] was performed for publications from January 2012 to April 2023 (Fig. 1). The search was conducted in PubMed separately for ESTRO journals only with the string (“JOURNAL_NAME”[jour]) AND (questionnaire or survey), where JOURNAL_NAME was “Radiother Oncol”, “Clin Transl Radiat Oncol”, “Phys Imaging Radiat Oncol” or “Tech Innov Patient Support Radiat Oncol”. Inclusion criteria encompassed papers using questionnaires as the primary methodology to address a research question. Exclusion criteria were studies using or developing patient questionnaires, Delphi consensus studies, surveys for consensus-seeking or guideline creation, surveys on quality assurance, clinical trial site feasibility/selection, and audits. The search was limited to ESTRO journals as these guidelines are developed under the auspices of ESTRO, and the volume of relevant papers within these journals provided a sufficient basis for the guideline development.

The screening process was collaboratively performed by five authors consulting each other for disagreement resolution, and all removed studies were verified by a second reviewer. Data extracted and analysed from the studies included information on the survey platform, the number of questions, response rate, dissemination, ethical considerations, handling of duplicate and incomplete responses, analysis, and whether the questionnaire was available to readers (as an appendix or part of the main manuscript). Additional data was collated, including geographical location, professional group, demographics, validity assessments, pre-testing and piloting, dissemination methods, response rate reporting, reporting practices, and terminology. The results were discussed among all authors. A formal quality assessment of the questionnaires was not undertaken.

## 3 Results

Fifteen general guidelines on survey-based research methodology were identified and reviewed (Appendix A.4). Eight guidelines applied to the broader field of medicine [22], [25], [26], [27], [28], [29], [30], [31], whereas others were developed specifically for the fields of surgery [32], [33], [34], internal medicine [35], dermatology [36], anaesthesiology [30] or paediatric urology [37]. The literature search on RO-specific survey studies yielded 182 articles. After full-text screening and application of in- and exclusion criteria, 84 remained eligible for analysis (Appendix A.5).

For design and data collection, 19/84 (23 %) surveys used SurveyMonkey, and 4/84 (5 %) Google Forms; the remainder did not specify the system that was used. Alternatives like Microsoft Forms and Qualtrics could also be considered. The number of questions in the surveys ranged from 7 to 135. Response rates were reported in 38/84 (45 %) studies and varied between 17 % and 100 %. Fifty/84 (60 %) surveys were disseminated on an international level. Ethical approval of the survey was mentioned in 13/84 (15 %) of the papers. Handling of duplicate respondents was only mentioned in 17/84 (20 %) of the papers; handling of incomplete data was only mentioned in 3/84 (4 %) papers. Forty-seven/84 (56 %) papers did not specify how the results were analysed. The questionnaires of the survey were available to the readers in 50/84 (60 %) of the papers.

Findings from 15 general guidelines and 84 RO-specific survey studies were used to complement the authors' knowledge and were synthesized into the following practical guidelines.

### 3.1 Questionnaire design

The research question should be clearly defined, and the decision to use a survey to answer it should be deliberate. The scope of the research question is an important consideration because cross-sectional studies imply one survey time-point, whereas longitudinal studies require repeated surveys [35]. The questionnaire design necessitates meticulous consideration of key elements: instructions, question-and-answer phrasing (especially for sensitive topics), response format, and overall length. The questionnaire design process should follow an intentional and methodological approach [22], [28], [34] aligned with the rigorous standards of quantitative and experimental methodologies traditionally applied in RO research.

The initial step involves the identification of constructs, representing ideas and concepts that cannot be directly quantified but are pertinent to the research question. The formulation of these constructs, along with specific questions, can be achieved through an explorative process that may involve a literature review, (Delphi) consensus, or focus groups [28]. Recent RO surveys from ESTRO Physics workshops showcase this approach. Workshops fostered expert discussions, pinpointing key constructs on specific themes for questionnaire development [2], [4], [38].

A thorough literature review can be conducted to determine if similar questions have been addressed previously and if existing questionnaires are suitable to answer the research question. In certain instances, validated instruments from previous research can be adopted [22], [34] as exemplified in RO research [11], [12], [39], [40], [41], [42]. In such cases, researchers should consider the applicability of these instruments to the target population [34].

This guideline focuses on the development of bespoke RO questionnaires. It presents a compendium of best practices regarding general phrasing (Table 1), question and answer type (Table 2), and logical structuring of the overall questionnaire (Table 3) and complements it with examples.

**Table 1:** Best practice for general phrasing of questions/responses.

**Questionnaire design**	**Examples**	**Comment**
Focus on a single construct, 20 words or fewer and easy to understand. Avoid jargon and complex terminology	Say:	Consider how non-native speakers might interpret the language, particularly when using specialist terms.
	“*Does your department use lung shielding for TBI to reduce the lung dose below the prescription dose?”*	
	Instead of:	
	*“Dose homogeneity and OAR shielding are crucial in TBI. Aside from build-up screens, arms shielding, solid water blocks and rice bags, does your department use additional lung shielding?”*	
Avoid double-barrelled questions that ask two things at once but rather ask two separate questions or choose the most important one	Say:	
	*Q1. “Does your department do brachytherapy?“*	
	*Q2. “Does your department do intraoperative radiotherapy?“*	
	Instead of:	
	*Q1. “Does your department do brachytherapy and intra-operative RT?* “	
Avoid absolute terms like “always”, “never”, “all”, “none”	Say:	Respondents may or may not consider exceptional cases in “always” and “never”.
	*“Does your department do adaptive RT?”*	
	Instead of:	
	*“Does your department always do adaptive RT?*”	
Use positively phrased questions	Say:	“un-” and “not” are easily missed when reading questions quickly. Positively phrased questions are quicker to read and understand.
	*“Do you systematically record patients’ quality of life?”*	
	Instead of	
	“*Do you****not****systematically record patients’ quality of life?”*	
Questions asking to rank items or asking for opinions should be phrased neutrally and in a non-leading way	*Say:*	
	*“How would you rate the potential benefit of proton therapy compared to photon therapy? “*	
	Instead of:	
	*“Do you agree that proton therapy is a better therapeutic option than photon therapy? “*

**Table 2:** Best practice and example for specific question and answer types.

**Questionnaire design**	**Examples **	**Comment**
Demonstrative questions followed by binary answers	*“Does your department have a dedicated MRI scanner (choose one answer only)?”* □ Yes □ No	Data can be presented as a simple percentage.
Ordinal and Likert scale	*“Are you satisfied with the staffing levels at your departments?”* Say: 1. Not at all satisfied 2. Somewhat satisfied 3. Moderately satisfied 4. Quite satisfied 5. Extremely satisfied Or with neutral option 1. Not at all satisfied 2. Somewhat satisfied 3. Neutral 4. Very satisfied 5. Extremely satisfied Instead of: *From 1 (not at all satisfied) to 5 (extremely satisfied)*	Consider if a neutral option is needed/wanted or not. Note that when unsure, a responder may select the neutral option, thus skewing the responses. Use verbal labels (easily interpretable) rather than only numbers where possible. The number of responses should be between 5 and 7. < 5 decreases the reliability of answers;, > 7 does not enhance reliability but overburdens respondents. Data can be presented in bar or pie charts.
Intervals	Say: *“How many years of experience do you have in the field of RO (post qualification/specialization or post-PhD)?”* • 0–4 • 5–9 • 10 or more Instead of: • 1–2 • 2–3 • 3–4 • 5–20 • 20 or more	Define non-overlapping intervals with reasonable and pertinent ranges. Data can be presented as bar charts.
Nominal questions with only one possible response	Say: *“For which treatment site do you wish to prioritise implementation of adaptive radiotherapy (choose only one)* • Head and neck • Lung • Prostate • Pancreas • Other (please specify)	Use survey options allowing only one answer (“Radio button” on most platforms.) Using only one response allows a percentage of responses which add up to 100 %. The question should be unambiguous (i.e., only one option is possible, here ensured by “prioritise”).
Nominal response with multiple choices possible	Say: *“In your department, which scans are routinely given to cervical cancer patients (multiple options can be selected)”:* • CT • MRI • PET • Other (please specify) Instead of: *“What kind of scanning do cervical cancer patients receive in your department?”*	Use survey options that allow multiple answers (“Check boxes” on most platforms). Data can be presented in absolute numbers using bar charts.
Open questions with a free text response	Say: “What additional information would you like to provide?”	Difficult for quantitative analysis. However, it can be useful for final comments to capture additional information. Open questions should generally be limited to 2–3 per questionnaire, preferably towards the end.

**Table 3:** Best practice for the general organisation of the questionnaire.

**Recommendation**	**Comment**
Start with a cover page clearly indicating the target audience, how results will be used, anonymization and data use	See template (Appendix A.1)
Questions are numbered and clearly organized by domain	When starting a new section, a brief introduction can be used. E.g. “This section includes questions on your department organization. The aim is to understand if there is a link between the departmental organization and the implementation of total body irradiation”
Within one domain, start with general questions, followed by more specific questions	General questions may also be used to determine whether the following questions are applicable. In this case, branching features can be used to tailor the survey process to the respondent.
Easier questions at the beginning, more difficult ones at the end	Respondents may stop answering when difficult questions arise. Starting with easy questions ensures some partial responses will be received.
Demographics and sensitive questions at the end	Evaluate the necessity of questions on protected characteristics unless relevant to the study (e.g. age, disability, gender, partnership and marriage, race, pregnancy and parental status, religion and belief, sex or sexual orientation) Unless necessary, make these questions optional.
Include definitions before potentially ambiguous questions	In RO, some terminology is used interchangeably, e.g. “shielding” in TBI: it may be considered to mean reducing the dose below the prescription level, reducing the dose to the prescription level, or as a build-up screen.
Clearly indicate when questions are compulsory or optional	If the survey system provides options for compulsory or optional settings, use them accordingly.
Clearly indicate when only one or multiple options can be selected	If the survey system provides settings, use them accordingly.

When designing specific questions, prioritize brevity in the question text (e.g. 20 words or less), avoid absolute terms (e.g. “always” or “never”) or technical idioms. Specify perspectives and use positive wording to prevent misinterpretation [22], [28], [36]. Avoid double-barrelled items by either framing two distinct questions or selecting the most pivotal one [22], [36], [43]. Consider how responses will be analysed and presented early in the design process. Response formats may be closed or open, with closed questions being quicker to administer and analyse. In contrast, open questions may provide qualitative information useful for elaboration. However, it is essential to ensure open questions have a clear aim and are relevant to the research objectives. Remember that analysing responses can be time-consuming and unreliable without proper structure or coding methods. Thus, the number of open-ended questions should be limited to address only the most important points requiring in-depth analysis [28], [43]. Demonstrative questions often yield binary responses, while those involving ranking or opinions should be phrased neutrally [28]. Ensure close-ended questions offer logically ordered and mutually exclusive response options [33], [36]. Choose an appropriate number of response options: for scale responses, choose 5 to 7 response options, considering whether a neutral option is desired or not [22], [28], [43]. Use verbal labels without numbers for clarity [22].

Structure questionnaires to place general inquiries before specific ones on the same topic [33]. Ensure a user-friendly survey layout with clear instructions, numbered questions, and organized groups. Tailor surveys with branching or filters to prevent respondents from being asked irrelevant or non-applicable questions. Consider placing demographic questions at the end due to sensitivity [22], [33]. Handle sensitive topics carefully; consult an institutional review board if needed. Disclose sensitive topics upfront, allowing respondents to skip them [33].

Consider that respondents may be international and not native speakers of the survey language. Consider abbreviations, RO terminology and definitions whereby different terms may have different meanings (e.g. the term “shielding” elicited varied interpretations in a survey on total body irradiation practice [8]). In RO, some tasks are carried out by different staff groups in different countries. Questionnaires in English are often used in research, yet translated versions may help reach a broader pool of respondents [44].

Concerning demographic questions, attention should be brought to diversity, equity, and inclusion (DEI). If certain demographic questions are necessary in the design of the questionnaire, care must be taken to consider legally protected group identities such as age, disability, gender, genetic information, race, religion and belief, sex or sexual orientation. Collecting such sensitive information must be performed only if necessary to answer the research question and consider that respondents may not wish to disclose this information (i.e. these questions should not be mandatory and/or should include an option “prefer not to say”). In this case, anonymous questionnaires will be preferred, and confidentiality of the results and strict adherence to the General Data Protection Regulation (GDPR) must be ensured for all areas involved with data collection and storage. Consider referring to an ethics committee if there are concerns. Official entities such as the European Commission have legislation and DEI guidelines that are helpful to address these demographic questions [45]. If the research is conducted in an institution outside the EU, it may be subject to other legislation.

### 3.2 Validation

After creating the questionnaire, researchers should thoroughly pre-test and pilot-test it to collect validity evidence [25], [27], [32], [37]. It should be noted that a questionnaire itself cannot be considered “valid”, but the conclusions inferred from the survey results can be considered applicable to a specific population and at a given point in time [27]. Pre-testing and pilot-testing are two distinct steps. Both are important for validation.

In the pre-testing phase, initiate a review and revision of the individual questions with individuals belonging to the target population [28], [32], [34], during interviews or focus group discussions [46]. “Cognitive testing” can be used, where pre-testers describe their thought process while answering specific questions (e.g. through voice or video recording or in written form). Cognitive testing can be supplemented with probe testing by asking specific questions about the pre-testers’ thought processes or asking them to rephrase questions or answers in their own words. This process can identify if the questions are clearly understood, consistently in the same way by multiple respondents, and as intended by the researchers [27], [37], [46]. Another possibility is to calculate the content validity index (CVI) based on feedback from 5 to 15 experts [47], [10]. Experts rate each question's relevance and clarity using a Likert scale to calculate the CVI. The Item-Level CVI (I-CVI) is calculated by dividing the number of experts rating an item as highly relevant by the total number of experts. The Scale-Level CVI (S-CVI) averages all I-CVIs across items. A CVI score of 0.78 or higher for I-CVI and 0.90 or above for S-CVI is generally considered acceptable for content validation. Questions with low CVI scores should be revised or removed, with the process repeated for refinement. Pre-testing can be considered an integral part of question development and should be repeated as needed until the results are satisfactory. In RO, questions should be tested with responders from different centres and countries, including people with different native languages and backgrounds. Pre-test the questionnaire with appropriate RO professionals due to variations in terminology and staff groups. Different professionals may respond differently to the same questions [2].

The next step is pilot-testing to evaluate the entire survey process in a structured manner [28], [46], and assess questionnaire length, flow, and ease of administration. Use a small group of respondents who were not involved in the development of the questionnaire for pilot testing to measure time and internal consistency [28] (Table 4).

**Table 4:** Example questions for pilot testing.

**Pilot testing consideration**	**Sample questions**
Time to complete the survey	How long did it take to complete the survey?
	Did any section take longer than expected?
Question and answer clarity	Were any questions difficult to understand or confusing?
	Were the response options clear and relevant?
	Were there any terms or phrases that were unclear or needed further explanation?
Survey flow and design	Did the survey flow logically from one section to the next?
	Were any transitions between questions or sections confusing?
Content	Were there any important questions or topics that you felt were missing?
	Was the information provided sufficient to understand the purpose and context of the survey?
Overall impression	Did you find the survey easy or challenging to complete?
	Do you have any further suggestions?

A group of 5 experts may be sufficient for national, single-professional target groups, but the group will need to be extended to more pilot testers for international and/or multi-professional surveys to be representative of the target group [36]. At the pilot-testing stage, the data analysis approach (see 3.4.) should also be tested and validated [27].

Consider a statistical measure to assess the reliability of a survey, such as Cronbach's alpha [48], [49]. It can evaluate the internal consistency or reliability of a set of items in the survey, thus indicating how well these items measure a single underlying construct. A Cronbach's alpha score between 0.7 and 0.9 is considered acceptable, suggesting that the items have a high level of agreement and cohesiveness. A low alpha score may indicate that some items do not align well with the construct being measured, prompting further review and revision of the questionnaire to improve its reliability and validity.

### 3.3 Dissemination

The target population must be clearly defined alongside the questionnaire design. Options can include individuals, RO departments, national scientific societies or health authorities. This should be clearly indicated in the cover letter (Appendix A.1). Whenever possible, restrict the survey distribution to the identified target group. Use a structured database for efficient dissemination, especially through scientific or professional societies’ databases with filtering capabilities.

If the survey is distributed by a scientific society the preferred means of distribution may be e-mailing to a large distribution list. For ESTRO, member information, including profession, experience, department, hospital, country of work, and main field of expertise, can facilitate targeted survey dissemination, reaching only relevant groups and thereby reducing survey fatigue. A well-curated mailing list also minimizes the need for extensive demographic questions. ESTRO members wishing to conduct a survey endorsed by ESTRO may use the ESTRO SurveyMonkey account and coordinate dissemination with the ESTRO office. In this case, the data is stored on the password-protected ESTRO account. The collection of IP addresses is optional and should generally be turned off unless necessary and justified (in this case, it should be indicated in the cover letter). Individual responses and summary data can be exported manually for analysis. The researchers are responsible for handling the data with care and confidentiality. Social media can also be used for dissemination. Consider appointing national coordinators, especially in international surveys, to improve response rates [50], [51]. Where relevant to the research question, make efforts to reach smaller clinics in middle or low-income countries, as RO researchers have shown that academic centres and public clinics in high-income countries were more likely to respond [2], [38]. Identifying contacts from these clinics will assist with this.

A template cover letter is available in Appendix A.1. It should include:

1. The research problem, context, and purpose of the survey
2. Description of the target group and rationale for the importance of the study and participation
3. Confidentiality details, including response storage and access conditions, if applicable, and explanation of data usage
4. Provision of contact details for potential respondents to ask further questions.
5. Information on how to access survey findings and whether demographic questions will be included
6. Indication of the estimated time needed for survey completion
7. Including the logo of the organization that is distributing the survey may help increase the response rate

It is recommended to send a maximum of three reminders [52], [53], [54], [55]. Studies suggest that this quantity effectively boosts an invitee’s inclination to participate. Additionally, previous research underscores the importance of considering the optimal timing for follow-ups based on the anticipated survey duration [55]. Their findings align with the notion that the shorter the expected survey duration, the fewer reminders are necessary, and the intervals between them should be condensed. For online surveys, initiating early reminders approximately three or four days after the survey launch appears to be an optimal strategy. This holds true for multiple reminders dispatched within a concise three-day timeframe [52], [53], [54], [55].

### 3.4 Analysis

Ensure the involvement of appropriate experts, such as those for qualitative analysis and statisticians, as needed for data analysis. Consider the type of analysis during the design stage and validate it accordingly. A well-designed survey should ensure straightforward analysis. Data analysis for survey-based research involves specific tasks, including calculating response or completion rates, managing missing or incomplete data, handling duplicate responses, and analysing the collected questionnaire data. Before beginning data analysis, a data curation process should verify the presence of missing or duplicate responses and confirm that responses align with the intended criteria (e.g. one per institution).

Response rate, defined as the number of responses received divided by the total number of eligible respondents, is an important measure to estimate the reliability and representativeness of the collected data. This may be relatively easy to calculate for institutional surveys or well-defined target groups when the survey is distributed to a well-curated mailing list. In RO surveys, responses vary with some studies achieving a rate > 90 %. Such high response rates could be attributed to following up non-responders [1], [56], [57], [58]. Ascertaining reasons for non-response is also good practice [59]. The total number of eligible respondents is difficult to assess in certain cases. For example, when the mailing list cannot be refined to include only the target population, the maximum possible number of responses is unknown. Response rate is particularly difficult to calculate for web-based surveys disseminated via social media or advertised at congresses and events. In such instances, completion rates may be a more appropriate measure [25]. Particularly if response rates are low, investigate potential differences between respondents and non-respondents to characterize the possibility of bias or non-generalizable conclusions [28] and note low response as a limitation. As an example, Gasnier *et al.* compared the number (and demographics) of their respondents to that of the ESTRO membership [11]. For institutional surveys, resources like the DIRAC database or the PTCOG list of proton therapy centres can be used to assess global response rates [1], [2], [38], [58].

In curating the data, duplicate responses must be addressed, particularly when a questionnaire is directed towards an institution. Consider how this will be done before questionnaire responses are reviewed to avoid bias. Responses from different individuals at the same institution can be either combined or ignored based on similarity or contradiction [4], [60] and an expert researcher should be consulted. If one person provides multiple responses, the latest valid response should be retained.

Partially completed questionnaires can be handled by using all available responses for each question in descriptive analyses and stating the absolute number (and percentage) of responses considered for each question. If an online survey is administered, partially completed responses can be eliminated using mandatory questions and answers; however, mandating all responses may lower the completion rate.

For data analysis itself, the type of statistical analysis will depend on the research question and the type of data collected [61]. Descriptive statistics can be used to estimate population parameters and describe associations. Ordinal and categorical data items should be summarised using frequencies, medians and/or modes, and denominators should be reported when percentages are used. Avoid combining categories or dichotomising data for analysis to prevent information loss. Explore associations between ordinal variables using non-parametric rank-based tests (e.g. Kendals Tau correlation coefficient). If responses to questions are to be reported in subgroups e.g. by demographics type factors, avoid “over-filtering” the data such that the subgroups become too small to draw conclusions.

In RO surveys, analytical approaches vary widely. For example, population parameters can be described by reporting percentages of centres utilizing an investigated facet, the involvement of various staff groups in this facet, and the time dedicated to this facet across surveyed centres over a given period [3]. Bertholet *et al.* described associations by investigating professional practice changes during the COVID-19 pandemic for different clusters based on the daily number of infections in the country of the respondents [62].

Consider the details to include in the analysis of the responses, i.e. descriptions only [53] or a more comprehensive statistical analysis [42], [63], [64]. This will depend on the research question and data collected and should be clearly set out in the methodology. It is possible to associate clinical practices with professional factors, e.g. assessing if the responder works in an academic environment and dedicates over 50 % of their work to clinical tasks [65]. Walker *et al.* used logistic regression to identify domains associated with the high adoption of radiotherapy in the treatment of muscle-invasive bladder cancer [66]. For longitudinal studies, i.e. when repeating the survey over time or when comparing data pre- and post-intervention, apply appropriate analytical methods to assess trends over time (e.g. regression or ANCOVA).

### 3.5 Reporting

When reporting and detailing the survey results, consider structuring the result section to follow the project's objectives, themes, and main messages rather than by question order. It is also important to double-check whether the reported results cover all the starting research objectives. Focus on the survey's target audience and frame the reporting, considering that the relevant information should be appropriately conveyed to the key stakeholders. Reporting, like analysis (section 3.4), should be considered during questionnaire design.

Transparent and comprehensive reporting of survey studies is essential and should follow general guidelines like the “Checklist for Reporting Results of Internet E-Surveys” (CHERRIES) [25] or “Consensus-Based Checklist for Reporting of Survey Studies“ (CROSS) [35]. The questionnaire should be included in the publication as part of the main article or supplementary material.

Using graphical summaries to visualize the data (e.g. bar charts for comparing categorical data, pie charts for percentages, and histograms to display the distributions) can be helpful in focusing on key data. They should be crafted as self-explanatory items since the reader may access them independently from consulting the main text. Avoid overly complex 3D graphics and purposefully use colour/shading/patterns. Results from binary questions can be simply reported as percentages, while results for most closed question types (Likert, nominal, interval) can be effectively presented in bar charts (Table 2). In tables and figures, indicate how many responses the results have been calculated with “n = x”. Consider reporting both the absolute number of responses and the percentage of total answers in the results (e.g. 30/60 respondents (50 %) use C-arm linear accelerators for SBRT). Especially when there are only partial responses, the total number of responses received for each question should be reported to put percentages into perspective.

When conducting sub-group analysis, researchers should evaluate whether the results are important enough to be included in the main manuscript or if they are better suited for supplementary material. If the analysis reveals statistically significant findings, it is crucial to assess whether these findings have practical or clinical relevance before deciding to highlight them.

Often, survey data highlight the correlation between variables. Consequently, survey-based research can rarely be used to conclusively prove causality. This should be considered when reporting on correlation data and discussing messages to convey to the reader. Finally, exercise careful consideration of the limitations of the study, particularly the use of a survey as a methodological tool, when concluding based on survey findings. Report a thorough understanding of the survey's limitations and range of applicability to judiciously interpret and apply its outcomes.

## 4 Conclusion

In conclusion, this guideline is a comprehensive framework for conducting survey-based research in radiation oncology. It emphasizes the crucial need for methodological rigour at every stage of the research process—from survey design and validation to dissemination, analysis, and reporting. The scoping review highlighted some shortcomings in the current level of survey-based research papers with a minority of them reporting response rate, ethical approval, handling of duplicate or incomplete data, and data analysis. Following these guidelines allows researchers to maintain consistency and reliability in gathering insights from professionals and institutions involved in radiation oncology. This approach facilitates the advancement of knowledge within the field and supports evidence-based decision-making and quality improvement initiatives across diverse clinical settings and staff groups. Thus, this guideline is essential for promoting rigorous research standards and advancing the quality of research outcomes in the field of radiation oncology.
