# Supplementary material for: ESTRO guidelines for developing questionnaires in survey-based radiation oncology research
Source: Clin Transl Radiat Oncol. 2024 Nov 24;51:100895. doi: 10.1016/j.ctro.2024.100895 (PMC11786078; doi:10.1016/j.ctro.2024.100895)
Supplement: Supplementary Data 1 [file mmc1.docx]

**Appendices**

**Appendix A.1.** Template for cover letter and recurring questions.

The following is meant to be used as an example for a cover letter and as the first page of the survey. The elements in italics in square brackets indicate the purpose of each section. Some variations on the phasing are presented and should be selected depending on the purpose of the survey. When setting up the survey in ESTRO’s SurveyMonkey, account, ensure that IP addresses are not collected. In the case where collecting IP addresses is absolutely necessary and justified, this should be specified in the cover letter. Review the settings for data collection to determine whether respondents will be allowed to respond only once or more than once, if they can interrupt the survey and continue later, etc., and make this clear to the respondents.

Dear ESTRO Members,

*[Identifying the Problem]*

As dedicated professionals in the field of radiotherapy and oncology, you are acutely aware of the multifaceted impacts of climate change on public health and healthcare infrastructure. Climate change poses significant challenges, from increasing patient loads due to climate-related health issues to disrupting the availability of essential medical supplies.

*[Why respondents’ Participation Matters]*

Your insights and experiences are invaluable in addressing these pressing issues. By participating in this survey, you will provide critical information that reflects the unique perspectives and needs of ESTRO members. Your contributions will help us understand how climate change affects our profession and what actions are necessary to mitigate these effects.

*[How the Study Benefits You and Others]*

This study aims to enhance our collective response to climate change within the radiotherapy and oncology community. By participating, you help us gather essential data to inform policies and practices designed to safeguard our profession and improve patient care in the face of climate change challenges.

*[Social Usefulness of the Study (select what applies to your study)]*

The results of this survey will be disseminated in scientific journals and congress to inform the scientific/healthcare community and influence the standard of care/ future research/ development and implementation. The results will be shared with local and state officials, media outlets, healthcare organizations, and relevant professional groups. These findings will play a crucial role in shaping policy decisions and raising awareness about the specific impacts of climate change on healthcare. By contributing to this survey, you will contribute to advancing the field of climate change in radiation oncology research. You ensure that the voice of the ESTRO community is heard by key decision-makers and the public.

*[Confidentiality Assurance]*

We understand the importance of confidentiality. The survey is anonymous, and the data will be stored on the password-protected Survey Monkey account belonging to ESTRO. Your responses will be used in summary form only to protect your confidentiality. Your individual responses will not be identifiable in any reports or publications. Due to the anonymity of the responses, it is not possible to withdraw from the survey after it has been completed. *[Adjust if another format is used]*

*[Contact for Questions]*

Should you have any questions about the study, please feel free to contact *[Name of Contact Person]* at *[Email Address]* or *[Phone Number*]. We are here to provide any information or clarification you may need.

*[Access to Survey Results]*

If you would like to receive a summary of the survey results, please indicate your preference at the end of the survey. We will ensure that you receive a comprehensive overview of the findings.

*[Demographics Questions (if applicable)]*

Demographic questions are included at the end to identify possible differences between groups but are not mandatory. This option is included if you prefer not to answer any of these questions.

*[Time to answer the survey (select and adjust depending on the chosen data collection setting)]*

The survey should take you approximately 5 to 10 min to complete. You can interrupt and continue filling out the survey or modify your responses at any time if you use the same computer (depending on cookie settings) until you press the submit button. *Or* this link is personal to you, and you can reply only once. *Or* you fill out the survey multiple times to submit more than one response.

*[Appreciation for Your Participation]*

We deeply appreciate your time and effort in participating in this important survey. Your voice is crucial in building a sustainable and resilient future for all.

Thank you for your valuable contribution.

Sincerely,

*[Your Name]*

*[Your Title/Organization]*

The following gives examples of questions that can be included if relevant to the research question. Researchers are encouraged to choose the questions (s) depending on their relevance to their survey. Questions can be further adapted depending on the purpose of the survey. Many of these questions can be used for filtering or sub-group analysis; however, thought should be given to the categories that are of interest and if the questions are pertinent. These questions can also be used to compare your sample to the target size and evaluate the generalizability of the results (e.g. are the demographics of the responder representative of the ESTRO membership?)

Recommended demographics questions and answer choice:

What is your age?

- Under 18
- 18-24
- 25-34
- 35-44
- 45-54
- 55-64
- 65+
- Prefer not to say

What is your gender?

- Male
- Female
- Non-binary
- Gender non-conforming
- Prefer not to say
- Other (specify)

With a drop-down list (available in Survey Monkey help for example). Avoid asking where people are from, rather consider what location is relevant to the research question being addressed by the questionnaire. Consider:

- Where do you currently reside?
- In which country do you work?
- In which country did you receive your training?

What is your role?

- (Radio)biologist
- Clinical oncologist
- Computer scientist
- Dosimetrist
- Medical oncologist
- Medical physicist
- Radiation oncologist
- Radiation therapist/RTT
- Other (please specify)

Approximately what proportion of your working time is dedicated to (matrix question format):

|  | 10-24% | 25-49% | 50-74% | 75-100% |
| --- | --- | --- | --- | --- |
| Clinical work |  |  |  |  |
| (Academic/clinical) research |  |  |  |  |
| Teaching |  |  |  |  |
| Industry (incl. Research and development) |  |  |  |  |
| Administrative |  |  |  |  |

For questions related to centre size and patient volume. Here consider which measure(s) is/are most relevant to your research question:

Is your centre (select all that apply):

- Public
- Private
- Academic

How many patients are treated with external beam radiotherapy per year in your institution (approximately)

- Small volume <1000 patients/year
- Medium volume (1000-2000 patients/year)
- Large volume (> 2000 patients/year

How many treatment units (linear accelerators, brachytherapy afterloaders, proton treatment rooms, etc) are in service at your institution?

- 1-2
- 3-6
- 7-10
- 10+

**Appendix A.2.** Checklist for questionnaire design, validation, dissemination, analysis, and reporting

Questionnaire design

ם Literature search is done to check for similar surveys

ם Survey title is short and concise

ם Cover letter includes:

- the purpose of the survey,
- a description of the target group,
- an explanation of how the data will be stored and used. If anonymous, how the confidentiality of the responses will be guaranteed
- an indication of the estimated time needed for survey completion
- a contact email for further questions and the logo

ם Question formats are according to tables 1-3

ם Questions about name, institution, etc. are at the end

ם Diversity, equity and inclusion questions are handled in accordance with relevant legislation

ם Analysis determined prior to dissemination – including how to deal with unfinished and duplicate responses and bias, and involvement of a statistician if relevant

Validation

ם Pre-testing done

ם Pilot testing is done – including several relevant professional groups and nationalities if relevant

Dissemination

ם National coordinators appointed where relevant

Data analysis & reporting

ם CROSS and/or CHERRIES checklists were followed [25,35]

ם Overall number of responses is listed for each question

ם The questionnaire is included in the appendix/supplementary material

ם Conclusions are supported by the data and not overreaching (e.g. correlation and causation are distinguished)

**Appendix A.3.** Glossary of survey-based research terms

*Content validity index:* The degree to which an instrument has an appropriate sample of items for the measured construct and is an important procedure in scale development. The content validity index (CVI) is the most widely used index in quantitative evaluation.

*Cross-sectional survey:* An observational study design where the investigator measures the outcome and the exposures in the study participants at a specific time point.

*DEI:* Diversity, equity, and inclusion.

*Delphi consensus study:* A structured communication technique or method used to gain reliable expert consensus through systematic rounds of interviewing. Originally developed as a systematic, interactive forecasting method which relies on a panel of experts.

*Descriptive statistics:* Brief informational coefficients that summarize a given data set, which can represent the entire population or a sample of a population.

*DIRAC:* DIrectory of RAdiotherapy Centres is the world’s most comprehensive database on radiotherapy resources. It comprises current and historical global data on radiotherapy centres, teletherapy machines, brachytherapy units, treatment planning systems, computed tomography systems and simulators.

*The Dillman method:* A tailored design method conceived in the early 1970s as an approach to designing mail and telephone surveys that emphasized giving attention to all aspects of questionnaires and survey implementation procedures experienced by recipients of survey requests.

*GDPR:* General Data Protection Regulation.

*Longitudinal studies:* Studies that employ continuous or repeated measures to follow particular individuals over prolonged periods—often years or decades.

*Open question:* A question where participants answer in their own words.

*Closed questions:* A closed-ended question refers to any question for which a researcher provides research participants with options from which to choose a response.

*PRISMA:* Preferred Reporting Items for Systematic Reviews and Meta-Analyses.

**Appendix A.4.** List of publications included in the review of general survey-based research guidelines

| Title | Reference, year of publication | Field | Focus |
| --- | --- | --- | --- |
| Improving the quality of web surveys: The Checklist for Reporting Results of Internet E-Surveys (CHERRIES) | [25] Eysenbach G., Journal of Medical Internet Research, 6(3), 2004 | Medical (general) | Practical checklist, specific to internet surveys |
| A guide for the design and conduct of self-administered surveys of clinicians | [28] Burns KEA et al. Canadian Medical Association Journal, 179(3), 2008 | Medical (general) | Covers type of survey, design, testing administration, statistical testing, survey reporting. |
| A Consensus-Based Checklist for Reporting of Survey Studies (CROSS) | [35] Sharma A et al. Journal of General Internal Medicine, 36(10), 2021 | Internal Medicine | DELPHI-based consensus on reporting web- and non-web-based surveys |
| A brief guide to survey methodology for vascular surgeons | [32] Smeds MR. Seminars in Vascular Surgery, 35(4), 2022 | Vascular surgery | General recommendation and technical definition on survey methodology |
| Educator's blueprint: A how-to guide for survey design | [22] Hill J et al. AEM Education and Training, 6(4), 2022 | Medical Education | Survey development with practical examples |
| Educator's blueprint: A how‐to guide for collecting validity evidence in survey‐ based research | [27] Hill J et al. AEM Education and Training, 6(6), 2022 | Medical Education | Collecting validity evidence |
| Educator’s blueprint: A how-to guide for developing high-quality multiple-choice questions | [29] Gottlieb et al. AEM Education and Training, 7(1), 2023 | Medical Education | Development of multiple-choice questions |
| Educator’s blueprint: A how-to guide on survey administration | [26] Ogle KY et al. AEM Education and Training, 7(5), 2023 | Medical Education | Survey administration |
| Practical Guidelines to Develop and Evaluate a Questionnaire | [36] Kishore K et al. Indian Dermatology Online Journal, 12(2), 2021 | Dermatology | Development of questionnaires aimed at patients |
| AAPOR Reporting Guidelines for Survey Studies | [33] Pitt SC et al. JAMA Surgery, 156(8), 2021 | Surgery | Reporting guidelines with 12-item checklist |
| A guide to evaluating survey research methodology in pediatric urology | [37] Li B. Journal of Pediatric Urology, 17(2), 2021 | Pediatric Urology | Guide to survey evaluation with an example |
| Survey research in anesthesiology: a field guide to interpretation | [30] Schroek H et al. Regional Anesthesia and Pain Medicine, 45(7), 2020 | Anesthesiology | Practical framework for clinical interpretation of survey-derived outcomes |
| Practical Guide to Survey Research | [34] Brasel K et al. JAMA Surgery, 155(4), 2020 | Surgery | General guidelines |
| Good practice in the conduct and reporting of survey research | [43] Kelley K et al. International Journal for Quality in Health Care, 15(3), 2003 | Health care | Good practice in conducting and reporting for survey-based research |
| Reporting guidelines for survey research: an analysis of published guidance and reporting practices | [31] Bennett et al. PLoS Medicine, 8(8), 2010 | Medicine (general) | Reporting |

**Appendix A.5.** Included papers using survey-based methodology in the ESTRO journals

| Title | First author | journal | year | doi |
| --- | --- | --- | --- | --- |
| Learning radiation oncology in Europe: Results of the ESTRO multidisciplinary survey | Bibault JE | Clin Transl Radiat Oncol | 2018 | 10.1016/j.ctro.2018.02.001 |
| Cancer clinical trials - Survey evaluating patient participation and acceptance in a university-based Comprehensive Cancer Center (CCC) | Kessel KA | Clin Transl Radiat Oncol | 2018 | 10.1016/j.ctro.2018.10.001 |
| Helping patients make informed decisions. Two-year evaluation of the Gustave Roussy prostate cancer multidisciplinary clinic | Patrikidou A | Clin Transl Radiat Oncol | 2018 | 10.1016/j.ctro.2018.07.001 |
| The status of radiation oncology (RO) teaching to medical students in Europe | Ben Mustapha S | Clin Transl Radiat Oncol | 2019 | 10.1016/j.ctro.2019.04.010 |
| Patterns of practice in palliative radiotherapy for bleeding tumours in the Netherlands; a survey study among radiation oncologists | Strijbos J | Clin Transl Radiat Oncol | 2019 | 10.1016/j.ctro.2019.01.004 |
| A national perspective about the current work situation at modern radiotherapy departments | Lindberg J | Clin Transl Radiat Oncol | 2020 | 10.1016/j.ctro.2020.08.001 |
| Conducting research in Radiation Oncology remotely during the COVID-19 pandemic: Coping with isolation | Dhont J | Clin Transl Radiat Oncol | 2020 | 10.1016/j.ctro.2020.06.006 |
| Do radiation oncologists talk about sexual health and dysfunction with their cancer patients? Results of the igls-vienna-sexmed-survey | Bräutigam E | Clin Transl Radiat Oncol | 2020 | 10.1016/j.ctro.2020.01.005 |
| The potential role of MR-guided adaptive radiotherapy in pediatric oncology: Results from a SIOPE-COG survey | Seravalli E | Clin Transl Radiat Oncol | 2021 | 10.1016/j.ctro.2021.05.008 |
| A multi-centre survey reveals variations in the standard treatments and treatment modifications for head and neck cancer patients during Covid-19 pandemic | Vasiliadou I | Clin Transl Radiat Oncol | 2021 | 10.1016/j.ctro.2021.06.002 |
| Impact of transitioning to an online course - A report from the ESTRO gyn teaching course | Tan LT | Clin Transl Radiat Oncol | 2021 | 10.1016/j.ctro.2021.06.001 |
| European radiation oncology after one year of COVID-19 pandemic | Slotman BJ | Clin Transl Radiat Oncol | 2021 | 10.1016/j.ctro.2021.03.011 |
| Prophylactic cranial irradiation in patients with small cell lung cancer in The Netherlands: A population-based study | Tomassen ML | Clin Transl Radiat Oncol | 2021 | 10.1016/j.ctro.2021.02.001 |
| A year of pandemic for European particle radiotherapy: A survey on behalf of EPTN working group | Barcellini A | Clin Transl Radiat Oncol | 2022 | 10.1016/j.ctro.2022.02.004 |
| Hypofractionated radiotherapy combined with targeted therapy or immunotherapy: Dutch survey on current practice, knowledge and challenges | van Aken ESM | Clin Transl Radiat Oncol | 2022 | 10.1016/j.ctro.2022.01.002 |
| Prospective assessment of stress and health concerns of radiation oncology staff during the COVID-19 pandemic | Christ SM | Clin Transl Radiat Oncol | 2022 | 10.1016/j.ctro.2022.06.001 |
| A year of pandemic for European particle radiotherapy: A survey on behalf of EPTN working group | Barcellini A | Clin Transl Radiat Oncol | 2022 | 10.1016/j.ctro.2022.02.004 |
| Current practices and perspectives on the integration of contrast agents in MRI-guided radiation therapy clinical practice: A worldwide survey | Boldrini L | Clin Transl Radiat Oncol | 2023 | 10.1016/j.ctro.2023.100615 |
| Prospective Pilot study of Quality of Life in patients with severe late-radiation-toxicity treated by Low hyperbaric-oxigen-therapy | Vera-Rosas A | Clin Transl Radiat Oncol | 2023 | [10.1016/j.ctro.2023.100620](https://doi.org/10.1016/j.ctro.2023.100620) |
| Professional practice changes in radiotherapy physics during the COVID-19 pandemic | Bertholet J | Phys Imaging Radiat Oncol | 2021 | 10.1016/j.phro.2021.06.002 |
| Inter-centre variability of CT-based stopping-power prediction in particle therapy: Survey-based evaluation | Taasti VT | Phys Imaging Radiat Oncol | 2018 | 10.1016/j.phro.2018.04.006 |
| Machine learning applications in radiation oncology: Current use and needs to support clinical implementation | Brouwer CL | Phys Imaging Radiat Oncol | 2020 | 10.1016/j.phro.2020.11.002 |
| Professional quality of life and burnout among medical physicists working in radiation oncology: The role of alexithymia and empathy | Di Tella M | Phys Imaging Radiat Oncol | 2020 | 10.1016/j.phro.2020.07.001 |
| Adoption of respiratory motion management in radiation therapy | Burtona A | Phys Imaging Radiat Oncol | 2022 | 10.1016/j.phro.2022.09.003 |
| Multicenter comparison of measures for quantitative evaluation of contouring in radiotherapy | Gooding MJ | Phys Imaging Radiat Oncol | 2022 | [10.1016/j.phro.2022.11.009](https://doi.org/10.1016/j.phro.2022.11.009) |
| Clinical use and future requirements of relative biological effectiveness: Survey among all European proton therapy centres | Heuvhel L | Radiother Oncol | 2022 | 10.1016/j.radonc.2022.10.004 |
| Establishing a benchmark of diversity, equity, inclusion and workforce engagement in radiation oncology in Europe - An ESTRO collaborative project | Gasnier A | Radiother Oncol | 2022 | 10.1016/j.radonc.2022.04.011 |
| Brachytherapy training survey among radiation oncology residents in Europe | Sturzda A | Radiother Oncol | 2022 | 10.1016/j.radonc.2022.10.030 |
| Recommended first-line management of brain metastases from melanoma: A multicenter survey of clinical practice | Jablonska A | Radiother Oncol | 2022 | 10.1016/j.radonc.2022.01.037 |
| Clues to address barriers for access to proton therapy in the Netherlands | Thijssen SV | Radiother Oncol | 2023 | [10.1016/j.radonc.2022.11.021](https://www.sciencedirect.com/science/article/pii/S0167814022045844?via%3Dihub) |
| Tools for large-scale data analytics of an international multi-center study in radiation oncology for cervical cancer | Exker S | Radiother Oncol | 2023 | [10.1016/j.radonc.2023.109524](https://doi.org/10.1016/j.radonc.2023.109524) |
| Management of oligo-metastatic and oligo-recurrent cervical cancer: A pattern of care survey within the EMBRACE research network | Chopra S | Radiotherapy and Oncology | 2021 | 10.1016/j.radonc.2020.10.037 |
| Feasibility and impact of national peer reviewed clinical audits in radiotherapy departments | Vaandering A | Radiotherapy and Oncology | 2020 | 10.1016/j.radonc.2020.01.012 |
| Current practice in proton therapy delivery in adult cancer patients across Europe | Tambas M | Radiotherapy and Oncology | 2021 | 10.1016/j.radonc.2021.12.004 |
| Hypofractionated radiation therapy for breast cancer: Preferences amongst radiation oncologists in Europe - Results from an international survey | Ratosa I | Radiotherapy and Oncology | 2021 | 10.1016/j.radonc.2020.10.008 |
| Brachytherapy in Belgium in 2018. A national survey of the brachytherapy study group of the Belgian SocieTy for Radiotherapy and Oncology (BeSTRO) | Salembier C | Radiotherapy and Oncology | 2020 | 10.1016/j.radonc.2020.07.010 |
| Patterns of practice for adaptive and real-time radiation therapy (POP-ART RT) part I: Intra-fraction breathing motion management | Anastasi G | Radiotherapy and Oncology | 2020 | 10.1016/j.radonc.2020.06.018 |
| Quality of radiotherapy services in post-Soviet countries: An IAEA survey | Rosenblatt E | Radiotherapy and Oncology | 2018 | 10.1016/j.radonc.2018.03.028 |
| Alexithymia and professional quality of life in radiation oncology: The moderator effect of the professional profile | Franco P | Radiotherapy and Oncology | 2021 | 10.1016/j.radonc.2021.01.033 |
| Development of staffing, workload and infrastructure in member departments of the European Organisation for Research and Treatment of Cancer (EORTC) radiation oncology group | Willmann J | Radiotherapy and Oncology | 2021 | 10.1016/j.radonc.2020.11.009 |
| Towards homogenization of total body irradiation practices in pediatric patients across SIOPE affiliated centers. A survey by the SIOPE radiation oncology working group | Hoeben BAW | Radiotherapy and Oncology | 2021 | 10.1016/j.radonc.2020.10.032 |
| National societies' needs as assessed by the ESTRO National Societies Committee survey: A European perspective | Garibaldi C | Radiotherapy and Oncology | 2020 | 10.1016/j.radonc.2020.08.001 |
| A national survey on radiation oncology patterns of practice in Switzerland during the COVID-19 pandemic: Present changes and future perspectives | Achard V | Radiotherapy and Oncology | 2020 | 10.1016/j.radonc.2020.05.047 |
| Professional quality of life and burnout amongst radiation oncologists: The impact of alexithymia and empathy | Franco P | Radiotherapy and Oncology | 2020 | 10.1016/j.radonc.2020.05.017 |
| COVID-19 outbreak and cancer radiotherapy disruption in Italy: Survey endorsed by the Italian Association of Radiotherapy and Clinical Oncology (AIRO) | Jereczek-Fossa BA | Radiotherapy and Oncology | 2020 | 10.1016/j.radonc.2020.04.061 |
| Radiotherapy staffing in the European countries: final results from the ESTRO-HERO survey | Lievens Y | Radiotherapy and Oncology | 2014 | 10.1016/j.radonc.2014.08.034 |
| Recommended first-line management of brain metastases from melanoma: A multicenter survey of clinical practice | Jablonska PA | Radiotherapy and Oncology | 2022 | 10.1016/j.radonc.2022.01.037 |
| Brachytherapy for locally advanced cervical cancer: A survey of UK provision of care and support | Humphrey P | Radiotherapy and Oncology | 2021 | 10.1016/j.radonc.2021.03.007 |
| Hypofractionated radiotherapy in the real-world setting: An international ESTRO-GIRO survey | Rodin D | Radiotherapy and Oncology | 2021 | 10.1016/j.radonc.2021.01.003 |
| ESTRO ACROP guidelines for target volume definition in the thoracic radiation treatment of small cell lung cancer | Le Pechoux C | Radiotherapy and Oncology | 2020 | 10.1016/j.radonc.2020.07.012 |
| Patterns of practice for adaptive and real-time radiation therapy (POP-ART RT) part II: Offline and online plan adaption for interfractional changes | Bertholet J | Radiotherapy and Oncology | 2020 | 10.1016/j.radonc.2020.06.017 |
| Organ at risk delineation for radiation therapy clinical trials: Global Harmonization Group consensus guidelines | Mir R | Radiotherapy and Oncology | 2020 | 10.1016/j.radonc.2020.05.038 |
| Exploring implementation of the ESTRO Core Curriculum at the national level | Giuliani M | Radiotherapy and Oncology | 2020 | 10.1016/j.radonc.2020.03.028 |
| The 2017 Assisi Think Tank Meeting on rectal cancer: A positioning paper | Valentini V | Radiotherapy and Oncology | 2020 | 10.1016/j.radonc.2019.07.001 |
| Liver phantom design and dosimetric verification in participating institutions for a proton beam therapy in patients with resectable hepatocellular carcinoma: Japan Clinical Oncology Group trial (JCOG1315C) | Nishio T | Radiotherapy and Oncology | 2019 | 10.1016/j.radonc.2019.06.005 |
| Interobserver variability in delineation of target volumes in head and neck cancer | van der Veen J | Radiotherapy and Oncology | 2019 | 10.1016/j.radonc.2019.04.006 |
| Consolidative thoracic radiotherapy in stage IV small cell lung cancer: Selection of patients amongst European IASLC and ESTRO experts | Putora PM | Radiotherapy and Oncology | 2019 | 10.1016/j.radonc.2019.02.010 |
| Patterns of proton therapy use in pediatric cancer management in 2016: An international survey | Journy N | Radiotherapy and Oncology | 2019 | 10.1016/j.radonc.2018.10.022 |
| Radiotherapy for recurrent prostate cancer: 2018 Recommendations of the Australian and New Zealand Radiation Oncology Genito-Urinary group | Lieng H | Radiotherapy and Oncology | 2018 | 10.1016/j.radonc.2018.06.027 |
| Development of quality indicators to monitor radiotherapy care for men with prostate cancer: A modified Delphi method | Tsiamis E | Radiotherapy and Oncology | 2018 | 10.1016/j.radonc.2018.04.017 |
| Practice patterns of image guided particle therapy in Europe: A 2016 survey of the European Particle Therapy Network (EPTN) | Bolsi A | Radiotherapy and Oncology | 2018 | 10.1016/j.radonc.2018.03.017 |
| Bladder-sparing radiotherapy for muscle-invasive bladder cancer: A survey of providers to determine barriers and enablers | Walker M | Radiotherapy and Oncology | 2017 | 10.1016/j.radonc.2017.08.036 |
| Patterns of care survey: Radiotherapy for women with locally advanced cervical cancer | de Boer P | Radiotherapy and Oncology | 2017 | 10.1016/j.radonc.2017.04.005 |
| Patient safety in external beam radiotherapy, results of the ACCIRAD project: Current status of proactive risk assessment, reactive analysis of events, and reporting and learning systems in Europe | Malicki J | Radiotherapy and Oncology | 2017 | 10.1016/j.radonc.2017.02.016 |
| Radiotherapy equipment and departments in the European countries: final results from the ESTRO-HERO survey | Grau C | Radiotherapy and Oncology | 2014 | 10.1016/j.radonc.2014.08.029 |
| Quality assurance standards drive improvements in the profile of radiation therapy departments participating in trials of the EORTC Radiation Oncology Group | Grant W | Radiotherapy and Oncology | 2014 | 10.1016/j.radonc.2014.09.003 |
| Guidelines for equipment and staffing of radiotherapy facilities in the European countries: final results of the ESTRO-HERO survey | Dunscombe P | Radiotherapy and Oncology | 2014 | 10.1016/j.radonc.2014.08.032 |
| Patient safety in external beam radiotherapy - guidelines on risk assessment and analysis of adverse error-events and near misses: introducing the ACCIRAD project | Malicki J | Radiotherapy and Oncology | 2014 | 10.1016/j.radonc.2014.08.011 |
| Planning of radiotherapy capacity and productivity | Slotman BJ | Radiotherapy and Oncology | 2013 | 10.1016/j.radonc.2013.02.006 |
| ESTRO ACROP guidelines for positioning, immobilisation and position verification of head and neck patients for radiation therapists | Leech M | Tech Innov Patient Support Radiat Oncol | 2017 | 10.1016/j.tipsro.2016.12.001 |
| Diversity in radiation therapist/therapeutic radiographer (RTT) advanced practice (AP) roles delivering on the four domains | Duffton A | Tech Innov Patient Support Radiat Oncol | 2021 | 10.1016/j.tipsro.2021.02.003 |
| The sustainability of the New Zealand radiation therapy workforce: Factors that influence intent to leave the workplace and profession | Taylor MR | Tech Innov Patient Support Radiat Oncol | 2020 | 10.1016/j.tipsro.2020.11.002 |
| International survey; current practice in On-line adaptive radiotherapy (ART) delivered using Magnetic Resonance Image (MRI) guidance | McNair HA | Tech Innov Patient Support Radiat Oncol | 2020 | 10.1016/j.tipsro.2020.08.002 |
| Radiation therapy technologists' involvement and opinion in research: A national survey in Italy | Piro D | Tech Innov Patient Support Radiat Oncol | 2020 | 10.1016/j.tipsro.2020.05.002 |
| Harmonization of breast cancer radiotherapy treatment planning in the Netherlands | Hurkmans C | Tech Innov Patient Support Radiat Oncol | 2021 | 10.1016/j.tipsro.2021.06.004 |
| Barriers and facilitators to the adoption of artificial intelligence in radiation oncology: A New Zealand study | Victor Mugabe K | Tech Innov Patient Support Radiat Oncol | 2021 | 10.1016/j.tipsro.2021.03.004 |
| The role of alexithymia and empathy on radiation therapists' professional quality of life | Franco P | Tech Innov Patient Support Radiat Oncol | 2020 | 10.1016/j.tipsro.2020.07.001 |
| Cautiously optimistic: A survey of radiation oncology professionals' perceptions of automation in radiotherapy planning | Batumalai V | Tech Innov Patient Support Radiat Oncol | 2020 | 10.1016/j.tipsro.2020.10.003 |
| Take Action Protocol: A radiation therapist led approach to act on anatomical changes seen on CBCT | Buijs M | Tech Innov Patient Support Radiat Oncol | 2021 | 10.1016/j.tipsro.2020.12.001 |
| Future-proof Radiation therapist (RTT) practice in a pandemic - Lessons learnt from COVID-19 | Kearney M | Tech Innov Patient Support Radiat Oncol | 2021 | 10.1016/j.tipsro.2021.02.001 |
| Intravenous contrast media in radiation therapy planning computed tomography scans - Current practice in Ireland | Minogue S | Tech Innov Patient Support Radiat Oncol | 2019 | 10.1016/j.tipsro.2019.11.002 |
| Exploring radiation therapist education and training | Coffey M | Tech Innov Patient Support Radiat Oncol | 2022 | 10.1016/j.tipsro.2022.09.006 |
| Surface guided radiation therapy: An international survey on current clinical practice | batista V | Tech Innov Patient Support Radiat Oncol | 2022 | [10.1016/j.tipsro.2022.03.003](https://doi.org/10.1016/j.tipsro.2022.03.003) |
| A new wave of leaders: Early evaluation of the interdisciplinary Foundations of Leadership in Radiation Oncology (FLiRO) program | Turner S | Tech Innov Patient Support Radiat Oncol | 2022 | 10.1016/j.tipsro.2022.09.004 |
